# The relationship between pre-surgery self-rated health and changes in functional and mental health in older adults: insights from a prospective observational study

**DOI:** 10.1186/s12877-023-03861-x

**Published:** 2023-03-31

**Authors:** Eva F. Mennig, Sarah K. Schäfer, Gerhard W. Eschweiler, Michael A. Rapp, Christine Thomas, Susanne Wurm

**Affiliations:** 1 Department of Prevention Research and Social Medicine, Institute for Community Medicine, University Medicine Greifswald, Walther-Rathenau-Strasse 48, 17475 Greifswald, Germany; 2 Department of Geriatric Psychiatry and Psychotherapy, Klinikum Stuttgart, Priessnitzweg 24, 70374 Stuttgart, Germany; 3 Leibniz Institute for Resilience Research, Wallstrasse 7, 55122 Mainz, Germany; 4 Geriatric Center at the University Hospital Tübingen, University Hospital of Psychiatry and Psychotherapy Tübingen, Calwerstrasse 14, 72076 Tübingen, Germany; 5 Department of Psychiatry and Psychotherapy, University Hospital of Tübingen, Calwerstrasse 14, 72076 Tübingen, Germany; 6 Department of Social and Preventive Medicine, University of Potsdam, Am Neuen Palais 10, 14469 Potsdam, Germany

**Keywords:** Self-rated health, Functional health, Mental health problems, Older adults, Elective surgery, Longitudinal, Resilience

## Abstract

**Background:**

Elective surgeries are among the most common health stressors in later life and put a significant risk at functional and mental health, making them an important target of research into healthy aging and physical resilience. Large-scale longitudinal research mostly conducted in non-clinical samples provided support of the predictive value of self-rated health (SRH) for both functional and mental health. Thus, SRH may have the potential to predict favorable adaptation processes after significant health stressors, that is, physical resilience. So far, a study examining the interplay between SRH, functional and mental health and their relative importance for health changes in the context of health stressors was missing. The present study aimed at addressing this gap.

**Methods:**

We used prospective data of 1,580 inpatients (794 complete cases) aged 70 years or older of the PAWEL study, collected between October 2017 and May 2019 in Germany. Our analyses were based on SRH, functional health (Barthel Index) and self-reported mental health problems (PHQ-4) before and 12 months after major elective surgery. To examine changes and interrelationships in these health indicators, bivariate latent change score (BLCS) models were applied.

**Results:**

Our analyses provided evidence for improvements of SRH, functional and mental health from pre-to-post surgery. BLCS models based on complete cases and the total sample pointed to a complex interplay of SRH, functional health and mental health with bidirectional coupling effects. Better pre-surgery SRH was associated with improvements in functional and mental health, and better pre-surgery functional health and mental health were associated with improvements in SRH from pre-to-post surgery. Effects of pre-surgery SRH on changes in functional health were smaller than those of functional health on changes in SRH.

**Conclusions:**

Meaningful changes of SRH, functional and mental health and their interplay could be depicted for the first time in a clinical setting. Our findings provide preliminary support for SRH as a physical resilience factor being associated with improvements in other health indicators after health stressors. Longitudinal studies with more timepoints are needed to fully understand the predictive value of SRH for multidimensional health.

**Trial registration:**

PAWEL study, German Clinical Trials Register, number DRKS00013311. Registered 10 November 2017 – Retrospectively registered, https://www.drks.de/drks_web/navigate.do?navigationId=trial.HTML&TRIAL_ID=DRKS00013311.

**Supplementary Information:**

The online version contains supplementary material available at 10.1186/s12877-023-03861-x.

## Background

Self-rated health (SRH) is the individual perception and rating of one’s own state of health and a central aspect of health-related quality of life (e.g., [1]). It is well known from epidemiological data that SRH is a robust predictor of functional and mental health [2–4]. However, research on the predictive value of SRH and its potential bidirectional relationship with other health indicators in clinical contexts is scarce. Especially when a major elective surgical procedure—a typical health-related stressor in older age—is imminent, assessing SRH may represent an efficient opportunity to predict later adaptation processes with respect to functional and mental health.

This idea ties in with the increasing research interest into physical resilience to age-related health stressors [5]. In old age, many people undergo (elective) surgery, however, both research and clinical practice show that responses to the same health stressors are heterogeneous, with some individuals showing fast and full recovery and others experiencing permanent loss of functioning [6, 7]. These heterogeneous responses to stressor exposure are in line with findings from the broader field of resilience research examining various types of stressors [8, 9], leading to an outcome-based definition of resilient responses, that is, resilience can be viewed as the maintenance or quick regain of (multidimensional) health (i.e., physical, functional and mental health) after stressor exposure. This raises the questions of what kind of pre-stressor factors, so-called resilience factors or indicators, can be used to predict resilient outcomes [10]. Emerging research into physical resilience [11, 12] along with a broad range of research into psychological resilience, suggested that such factors may be of (neuro)cognitive, psychological, or physiological manner [10, 13]. Building on this idea, SRH may constitute such a pre-stressor resilience factor allowing for the prediction of more or less resilient responses to health stressors in later life. Beyond other resilience factors (e.g., optimism, self-efficacy), SRH may have the unique potential to integrate aspects of psychological, physical and functional health, and can be seen as patient-centered factor by capturing patients’ subjective view on their present health status [14]. Psychological resilience research provided ample evidence for resilience factors changing and interacting with health outcomes over time [9, 13]. Thus, it is crucial to examine the interplay between SRH and multidimensional health indicators (i.e., resilient outcomes) in the context of health stressors.

### Relation of self-rated health and other health indicators

#### Evidence from non-clinical samples

Although SRH is often assessed using only a single item by which people are asked to rate their current health status, population-based longitudinal studies showed that SRH is a unique and independent predictor of mortality beyond other medical, behavioral and psychological health indicators (e.g., [15–17]). Furthermore, SRH was also found to be a predictor of functional health. Fong and Kok [18], for example, reported longitudinal epidemiological data of older community-dwelling adults and pointed out that participants who reported “poor” or “very poor” SRH were over 2 or over 4 times, respectively, more likely to experience functional decline after 2 years than respondents with good SRH. The predictive power of SRH for loss of functional abilities and concomitant development of functional decline has also been demonstrated in earlier studies with longitudinal epidemiological data [19, 20].

Beyond its robust association with functional health, SRH was also found to be predictive for mental health problems like depressive disorders. For instance, fair and poor SRH at baseline were associated with a significantly increased risk of developing major depression in older adults with Type II diabetes over a 3-year period [21]. In another population-based longitudinal study, SRH was one of the most important predictors for depression in older adults [22].

However, studies investigating the predictive value of SRH for both physical functioning and mental health outcomes were mostly limited to unidirectional effects, thereby neglecting the inverse path from functional and mental health to SRH. This is particularly noteworthy as functional health and mental health were also found to predict SRH. For example, better functional health and the absence of depression at baseline were among the most important predictors of good SRH after 12 months in a group of community-dwelling older adults [23].

So far, little is known about the longitudinal bidirectional interplay between SRH and functional and mental health as only a small number of studies has examined their interaction yet. In a longitudinal study of middle-aged adults, SRH predicted depressive symptoms, but the contemporaneous effect of depressive symptoms on SRH was non-significant [24]. In older adults, Peleg and Nudelman [25] found evidence for bidirectional effects between SRH and depressive symptoms and Jones et al. [26] showed that levels of SRH and depressive symptoms developed over time depending on how the other variable changed. Moreover, in Liang et al. [27], age-related changes in SRH were significantly correlated with trajectories of functional health. These mostly large-scale epidemiological studies provided preliminary evidence for the bidirectional interplay between (changes of) SRH and (changes of) multidimensional health.

#### Evidence from clinical samples

Research using clinical samples was rarer and yielded more heterogeneous results. In a sample of older patients in private medical practices, poorer SRH predicted more severe functional disability after 12 months, but did not predict the onset of depressive disorders [28]. In contrast, in a longitudinal cohort study of primary care patients, patients with fair to poor SRH at baseline had a two times higher risk for the onset of major depressive disorder than those reporting good to excellent SRH [29].

Further studies with clinical samples pointed to the importance of SRH in the context of health-related stressors: After a cardiac event (e.g., acute myocardial infarction), anxiety and depression risk significantly increased 2–4 months and 6–12 months after the event in patients with poor SRH [30]. In line, poorer SRH 6 weeks after a major medical event (myocardial infarction, stroke, or hip fracture) predicted disability 6 months after the event in older adults [31]. Furthermore, results of studies investigating SRH after stroke yielded that poor SRH was associated with detrimental outcomes such as decreased functionality and more negative affective-emotional state [32]. Thus, findings from clinical research provided evidence for the predictive value of SRH for functional and mental health.

### Self-rated health – a potential physical resilience factor?

Research in clinical samples already pointed to the prominent role of SRH in the context of age-related health stressors providing preliminary support for studying SRH as potential physical resilience factor. However, due to the spontaneous onset of health stressors like stroke or myocardial infarction in these studies, pre-stressor data were not always available, which is crucial for studying the process of maintaining or regaining multidimensional health [10, 13]. By contrast, elective surgeries offer the opportunity to collect such pre-stressor data. Despite this conceptual advantage for studying resilient responses, elective surgeries represent one of the most common health stressors in later life, which is often associated with persistent changes in functional and mental health, making it an important target of research into physical resilience [6]. In Germany alone, more than one third of inpatients undergoing various surgical interventions are aged 70 years and older [33]. Even though functional and mental health are of major importance for older patients after surgery, many studies solely focus on physical health outcomes (e.g., [34, 35]). The small number of studies linking SRH and data of surgical patients demonstrated that SRH is a significant predictor of post-surgery physical, mental and social health after total joint replacement [14] and spinal stenosis surgery [36]. However, no study yet specifically examined pre-to-post-surgery changes of functional and mental health and none of the previous studies assessed the interplay between pre-surgery SRH, functional and mental health with changes in these health indicators.

### Study aim

The current study aimed at addressing this gap by examining the associations of pre-surgery health indicators (i.e., SRH, functional health and self-reported mental health) with changes in these indicators 12 months after a major age-related health stressor. So far, little is known on the inverse association, that is, the predictive value of other health indicators for (changes in) SRH or the bidirectional relationship between SRH and other health indicators. Thus, we were interested in the bidirectional interplay between SRH and other health indicators as well as their predictive value for multidimensional health changes. For the present study, we used longitudinal clinical data of older adults (≥ 70 years) who underwent elective surgical procedures. The key advantages of this dataset are its large sample size and the broad range of elective surgical interventions. Building on previous evidence, we expected to find a bidirectional interplay of health indicators. We hypothesized that pre-surgery functional health will predict 1) changes in functional health and 2) changes in SRH 12 months after the elective surgery. With respect to mental health, we expected pre-surgery mental health problems and SRH to predict changes in 3) mental health problems and 4) SRH, in each case, 12 months after the elective surgery. We hypothesized that 5) better pre-surgery functional and mental health will be associated with improvement of SRH. Most importantly, 6) we expected better pre-surgery SRH to be associated with improvements of functional and mental health.

## Methods

### Participants and design

Data for this study comes from the PAWEL study, which was conducted in five German medical centers in southwestern Germany and investigated whether a cross-sectoral and multimodal intervention can prevent delirium and postoperative cognitive decline (POCD) in patients 70 years or older undergoing elective surgery (details on the study aim and the intervention are described in the Supplementary Material as well as in Sánchez et al. [37] and Deeken et al. [38]). For the current study, we used data from the assessment before or at hospital admission (i.e., pre-surgery) and the 12 months follow-up assessment (i.e., post-surgery). Study data were collected and managed by using SecuTrial® electronic data capture tools hosted at the University of Potsdam. The PAWEL study was funded from 2017 to 2021 by the “Innovationsfonds des Gemeinsamen Bundesausschusses (G-BA)”. There were no ethical concerns regarding the assessment of SRH, functional and mental health problems by the Ethics Commission of the Faculty of Medicine of the Eberhard-Karls University and University Hospital Tübingen and by the Ethics Commission of the University of Potsdam that both approved the PAWEL study. All patients or their legal guardians gave informed consent to the use of data by scientists involved in the PAWEL study, also for secondary analyses.

### Inclusion and exclusion criteria

To be included in the study patients needed to be 70 years or older and have been scheduled for elective surgery (i.e., cardiac, thorax, vessels, proximal large joints or spine, genitourinary, abdominal, or general elective surgery procedures) with a planned cut-to-suture time of at least 60 min under general, spinal, or regional anesthesia. Due to the interest in postoperative delirium, also patients with mild dementia and frailty were included in the study if they were able to consent to the trial or a legal guardian provided informed consent.

Patients were excluded when they were undergoing emergency surgery, were unable to consent due to insufficient German language skills or if they were newly diagnosed with moderate or severe dementia (clinical assessment by study physicians; red flag: Mini Mental State Examination < 15 or Montreal Cognitive Assessment < 8 [39, 40]) and had no legal guardian. Due to the longitudinal design of the study, patients were excluded if survival expectancy was less than 15 months or they lived in great distance (≥ 120 km) from the study site. For the current study, patients were excluded if they did not complete the pre-surgery assessment of SRH, functional and mental health (*n* = 74). Moreover, patients were excluded when they deceased to follow-up (*n* = 19), resulting in 1,580 patients that were included in our analyses. This also included 185 individuals of the PAWEL-RISK sub-sample without 12 months post-assessment [41]. They were included in our analyses on the total sample to use all available information of the pre-surgery assessment but were excluded in our completers analyses. Recruitment or assessment of these patients did not differ from the PAWEL total sample.

### Measures

All self-report measures were assessed using paper-and-pencil questionnaires in attendance of a trained study team member. In case patients experienced difficulties with completing the self-report measures, they received help from the study team.

#### Self-rated health

Self-rated health was assessed using the first item of the Short-Form-Health Survey (SF-12; [42]). This item assesses general health (i.e., “In general, would you say your health is excellent, very good, good, fair, poor?”). Ratings range from “1 = excellent” to “5 = poor”. For the current study, the item has been recoded and higher scores indicate better self-rated health. Test–retest reliability for the self-rated health assessment of the SF-12 was found to be acceptable over one year (0.55; [43]). The necessary license for use of the SF-12 was obtained.

#### Functional health

As indicator of functional health, we assessed self-rated functional status as degree of independence using the Barthel Index [44], which was scored according to the Hamburg Classification Manual [45]. The Hamburg Classification Manual ensures a standardized use of the items in geriatric facilities in German-speaking countries. The scale ranges from 0 to 100, whereby 0 means total dependence and 100 complete independence. The Barthel Index was found to be reliable and valid [46] and test–retest reliability was good over 2–4 weeks (0.79; [47]). In the current study, a well-established German version [48] has been used that is not under license.

#### Mental health problems

Self-reported mental health problems were assessed using the 4-item Patient Health Questionnaire (PHQ-4; [49]). The ultra-brief 4-item self-report measure comprises two items of the Patient Health Questionnaire 2 (PHQ-2; [50]) to assess depressive symptoms and two items of the Generalized Anxiety Disorder 2 scale (GAD-2; [51]) to evaluate anxiety symptoms. All items are rated on a 4-point Likert scale ranging from “0 = not at all” to “3 = nearly every day”. Higher scores indicate more severe mental health problems. Both PHQ-2 and GAD-2 were found to show good test–retest reliability (≥ 0.79 over three weeks [52]). In the current study, a validated German version of the PHQ-4 is used [49] that is not under license.

As covariates, we include age, gender and education levels assessed according to International Standard Classification of Education (ISCED; [53]). As auxiliary variable, we included pre-surgery cognitive functioning as assessed using the Montreal Cognitive Assessment [54], with higher scores indicating better cognitive functioning.

### Analyses

All analyses were conducted using *RStudio* version 2022.02.3 [55]. To examine the nature of missing data patterns, Little’s missing completely at random test [56] was performed using the *naniar* package [57]. To further explore the nature of missing data, binary logistic regressions were performed to predict completer versus non-completer status. Moreover, *t*-test for independent samples and *χ*^*2*^ tests were used to compare completers and non-completers.

Simple pre-to-post changes were examined by means of paired *t*-tests. Bivariate latent change score (BLCS) models were used to further examine predictors of within-individual change rather than between-individual differences over time (see Supplementary Material for a detailed explanation of our model choice; [58, 59]). Before performing BLCS modeling, raw data was centered relative to mean baseline scores for the respective outcome to facilitate intercept interpretation. For BLCS modeling, we used the *lcsm* package [60], making use of the *lavaan* package [61] and the *semTool* package [62]. Models were estimated using maximum likelihood estimations with robust standard errors and scaled test statistics (MLR) to account for non-normal distributed data [63]. Model fit was assessed using the comparative fit index (CFI; good fit: > 0.95), standardized root-mean-square residuals (SRMR; good fit: < 0.08), and root mean square error of approximation (RMSEA; good fit: < 0.07; [64]). For BLCS modeling, the full information maximum likelihood (FIML) approach was used as a conservative approach to handle missing data when missing is not (completely) at random [65]. In contrast to multiple imputation approaches, FIML does not impute missing values but estimates population parameters by determining the value that maximizes the likelihood function based on available data. Multiple imputations and FIML were found to yield comparable results [66]. In line with recommendations, we included auxiliary variables (i.e., variables associated with missingness) in our model where applicable to increase the likelihood of missing at random [67].

Four BLCS models were contrasted (i.e., no coupling effects, two unidirectional coupling effects, bidirectional coupling effects; see Supplementary Material for a schematic illustration of the BLCS models). In the no coupling effect model, the bidirectional coupling effects of SRH and functional / mental health were fixed to zero. In the unidirectional models, only an effect from SRH on functional / mental health (or vice versa) was estimated freely, whereas the reciprocal association was fixed to zero. In the bidirectional model, all cross-variable associations <y estimated freely. Fit of nested models was compared using the ANOVA function of *R,* which is based on the Satorra-Bentler [68] χ^2^ difference (χ^2^_diff_) test. A significant χ^2^_diff_ test indicates meaningful differences in model fit. These tests were also used to compare the size of coupling effects within one model, e.g., to investigate whether the effect of pre-surgery SRH on changes in functional health at 12-months follow-up assessment was significantly different from the effect of pre-surgery functional health on changes in SRH. For this purpose, we constrained these paths to be equal and compared the model fit of this model with another model allowing them to vary freely, with significant differences in model fit indicating differential effect sizes of coupling effects. To illustrate changes and coupling effects from SRH to functional health or mental health and vice versa, we used the equations proposed by Jajodia [69] and calculated expected changes based on unstandardized estimates, while all other results are reported based on standardized coefficients.

## Results

### Sample characteristics

We included data of 1,580 patients, with 794 completing all measures. Mean age was 77.27 years (*SD* = 4.79; age range: 70–96 years) and 53.2% of the sample were male. Table 1 presents sample characteristics for the total sample and completers along with a comparison between completers and non-completers.

**Table 1:** Sample characteristics of the total sample and completers

	**Total sample** (*n* = 1,580)	**Completers** (*n* = 794)	**Completers vs. Non-Completers**
Age (*M* [*SD*], range)	77.27 (4.79) 70 – 96	76.96 (4.64) 70 – 91	*t*(1578) = 2.64, *p* = .008, Cohen’s *d* = 0.13
Gender (% men)	53.2	52.6	χ^2^ (1) = 0.18, *p* = .675, Cramer’s *V* = 0.01
**Education (%)**			
No school degree	3.2	2.6	χ^2^ (1) = 1.96, *p* = .375, Cramer’s *V* = 0.04
Primary school or lower secondary education	73.0	72.7	
Upper secondary education	21.8	22.7	
Other degree	2.0	2.0	
**Partnership (%)**			
Married or living with a partner	62.8	63.0	χ^2^ (1) = 0.53, *p* = .970, Cramer’s *V* = 0.02
Married but living separately from the spouse	6.2	6.2	
Divorced	5.4	5.7	
Widowed	22.3	22.29	
Single	5.4	2.9	
**Pre-Surgery Status (M [SD])**			
Cognitive functioning	23.25 (3.96)	23.99 (3.59)	*t*(1528.8) = -7.57, *p* < .001, Cohen’s *d* = -0.38
Frailty	3.48 (1.35)	3.40 (1.30)	*t*(1562.2) = 2.30, *p* = .021, Cohen's *d* = 0.06
**Study variables at baseline (** ***M*** ** [** ***SD*** **])**			
Self-rated health	2.70 (0.81)	2.72 (0.81)	*t*(1578) = -0.87, *p* = .382, Cohen’s *d* = -0.04
Functional health	93.23 (15.53)	94.26 (13.39)	*t*(1472.5) = -2.66, *p* = .008, Cohen’s *d* = 0.13
Self-reported mental health problems	0.50 (0.81)	0.47 (0.77)	*t*(1562.8) = 1.80, *p* = .073, Cohen’s *d* = 0.09

*Note*. Comparisons between completers and dropouts used *t*-tests (or Welch *t*-tests) and χ^2^ tests where appropriate. Cohen’s *d* is presented as standardized effect size measure for *t*-tests. Cramer's *V* is used as effect size measure for χ^2^ tests

### Proof of rationale: study intervention

The intervention group, that received a cross-sectoral and multimodal intervention to reduce the risk of delirium after their surgery, and the control group without such an intervention did not differ significantly in our study variables at pre-surgery and follow-up assessment neither in the total sample, *p* ≥ 0.296, nor in the subgroup of completers, *p* ≥ 0.307. Therefore, patients of both groups were equally included in our analyses.

### Missing data

Due to the substantial amount of missing data at 12-months follow-up assessment (lost to follow-up: 49.7% of patients), we performed Little’s missing completely at random test, which indicated that data was not missing completely at random, χ^2^(27) = 170.0, *p* < 0.001. Since there is no concrete test available for concluding on missing at random or missing not at random, we aimed at predicting non-completer status based on sociodemographic data and pre-surgery variables, finding that completer status was significantly predicted by being randomized to the intervention group, *OR* = 1.29, 95% CI [1.05, 1.58], *p* < 0.001, and better pre-surgery cognitive functioning, *OR* = 1.10, 95% CI [1.07, 1.14], *p* < 0.001. No other study variable showed a unique association with completion status. This model only accounted for 4.0% of the variance in completion status. Based on these findings and given the large number of non-completers, we decided to present results of analyses based on complete cases and using the total sample with FIML separately in our tables; for the latter models, we added intervention group and pre-surgery cognitive functioning as auxiliary variables.

### Pre-to-post surgery health changes

Table 2 presents means, standard deviations and bivariate correlations for all study variables. Simple paired *t*-tests provided evidence for an increase of SRH, *t*(793) = 4.15, *p* < 0.001, Cohen’s *d* = 0.15, and functional health, *t*(793) = 5.93, *p* < 0.001, *d* = 0.21, from pre-surgery to 12-months follow assessment, and decreases of mental health problems, *t*(793) = -6.83, *p* < 0.001, *d* = -0.24. BLCS models were used to further examine these health changes, that is, the relationships between changes in SRH and functional health (Model 1) and between changes in SRH and mental health problems (Model 2) for completers. Moreover, models were re-estimated using the total sample (Models 3 and 4; see Table 3).

**Table 2:** Raw means, standard deviations, baseline-to-follow-up changes, and Pearson correlations among study variables for completers and the total sample

	*Total sample*	*Completers*						
	*M (SD)*	*M (SD)*	1	2	3	4	5	6
1. Self-rated health (Baseline)	2.70 (0.81)	2.72 (0.81)	-	.37	.27	.23	-.31	-.22
2. Self-rated health (12-months follow-up)		2.85 (0.79)		-	.23	.31	-.23	-.32
Baseline vs. 12-months follow-up		*t*(793) = 4.15, *p* < .001, Cohen’s *d* = 0.15						
3. Functional health (Baseline)	93.23 (15.53)	94.26 (13.39)	.28		-	.48	-.22	-.14
4. Functional health (12 months follow-up)		96.79 (9.16)				-	-.13	-.15
Baseline vs. 12-months follow-up		*t*(793) = 5.93, *p* < .001, Cohen’s *d* = 0.21						
5. Mental health problems (Baseline)	0.50 (0.81)	0.47 (0.78)	-.33		-.24		-	.42
6. Mental health problems (12 months follow-up)		0.28 (0.60)						-
Baseline vs. 12-months follow-up		*t*(793) = -6.83, *p* < .001, Cohen’s *d* = -0.24						

*Note*. Correlations for completers are presented above the diagonal, correlations for the total sample are shown below the diagonal. These were limited to correlations that only involved pre-surgery assessments, in other cases, correlations were equal to those of the completers subsample. Paired *t*-tests presented in this table refer to baseline-to-follow-up comparisons based on the completers sample. Cohen’s *d* is presented as standardized effect size measure

All correlations were significant at *p* < .001

### Model 1: Self-rated health and functional health

Comparing a model with unidirectional coupling allowing for a path from pre-surgery SRH to changes in functional health but not vice versa, with a model without coupling, the former resulted in a significantly improved model fit, χ^2^_diff_(1) = 9.46, *p* = 0.002 (see Table 3). Also, a model with a unidirectional path from pre-surgery functional health to changes in SRH showed better fit than the no coupling model, χ^2^_diff_(1) = 16.47, *p* < 0.001. Comparing both unidirectional models to a bidirectional coupling model including paths from pre-surgery SRH and functional health to changes of both indicators, the latter yielded the best model fit, χ^2^_diff_(1) ≥ 8.35, *p*’s ≤ 0.004 (see Table 3), which was also supported by good fit indices. Comparisons based on the total sample yielded the same pattern of results favoring a model allowing for bidirectional coupling (see Supplementary Material for details).

**Table 3:** Summary of fit statistics for bivariate latent change score models for self-rated health, functional health, and self-reported mental health

							*RMSEA*	
		χ^2^	*df*	BIC	*CFI*	*SRMR*	Est	90% CI
Completers								
Model 1 SRH – FH	1.1 No coupling	41.11	8	20,014.72	.91	.045	.072	.054, .092
	1.2 Unidirectional coupling (SRH → FH)	31.44	7	20,008.69	.94	.038	.066	.047, .088
	1.3 Unidirectional coupling (FH → SRH)	25.91	7	20,002.11	.95	.032	.058	.038, .080
	1.4 Bidirectional coupling (FH ↔ SRH)^*^	17.48	6	19,997.45	.97	.026	.049	.027, .073
	Model 1.1 vs. Model 1.2	χ^2^_diff_(1) = 9.46, *p* = .002						
	Model 1.1 vs. Model 1.3	χ^2^_diff_(1) = 16.47, *p* < .001						
	Model 1.2 vs. Model 1.4	χ^2^_diff_(1) = 15.27, *p* < .001						
	Model 1.3 vs. Model 1.4	χ^2^_diff_(1) = 8.35, *p* = .004						
	Model 1.4 with fixed vs. free coupling parameters	χ^2^_diff_(1) = 8.27, *p* = .004						
Model 2 SRH – MHP	2.1 No coupling	40.55	8	11,195.55	.94	.039	.072	.050, .095
	2.2 Unidirectional coupling (SRH → MHP)	31.42	7	11,193.32	.95	.032	.066	.043, .091
	2.3 Unidirectional coupling (MHP → SRH)	23.52	7	11,186.11	.97	.027	.055	.031, .080
	2.4 Bidirectional coupling (SRH ↔ MHP)^*^	16.08	6	11,185.46	.98	.022	.046	.019, .074
	Model 2.1 vs. Model 2.2	χ^2^_diff_(1) = 8.97, *p* = .003						
	Model 2.1 vs. Model 2.3	χ^2^_diff_(1) = 17.99, *p* < .001						
	Model 2.2 vs. Model 2.4	χ^2^_diff_(1) = 15.89, *p* < .001						
	Model 2.3 vs. Model 2.4	χ^2^_diff_(1) = 7.24, *p* < .001						
	Model 2.4 with fixed vs. free coupling parameters	χ^2^_diff_(1) = 2.25, *p* = .134						
Total sample								
Model 3 SRH – FH	3.1 No coupling	36.76	8	46,140.36	.94	.041	.048	.035, .062
	3.2 Unidirectional coupling (SRH → FH)	25.38	7	46,133.74	.96	.034	.041	.027, .056
	3.3 Unidirectional coupling (FH → SRH)	21.83	7	46,128.74	.97	.024	.037	.022, .052
	3.4 Bidirectional coupling (FH ↔ SRH)^*^	12.35	6	46,123.93	.99	.019	.026	.007, .043
	Model 3.1 vs. Model 3.2	χ^2^_diff_(1) = 9.54, *p* = .002						
	Model 3.1 vs. Model 3.3	χ^2^_diff_(1) = 14.43, *p* < .001						
	Model 3.2 vs. Model 3.4	χ^2^_diff_(1) = 13.13, *p* < .001						
	Model 3.3 vs. Model 3.4	χ^2^_diff_(1) = 8.17, *p* = .004						
	Model 3.4 with fixed vs. free coupling parameters	χ^2^_diff_(1) = 12.21, *p* < .001						
Model 4 SRH – MHP	4.1 No coupling	41.06	8	32,534.08	.95	.033	.051	.036, .067
	4.2 Unidirectional coupling (SRH → MHP)	32.60	7	32,533.19	.96	.028	.048	.032, .066
	4.3 Unidirectional coupling (MHP → SRH)	24.06	7	32,525.49	.97	.023	.039	.023, .057
	4.4 Bidirectional coupling (SRH ↔ MHP)^*^	17.25	6	32,526.12	.98	.019	.034	.016, .054
	Model 4.1 vs. Model 4.2	χ^2^_diff_(1) = 8.49, *p* = .004						
	Model 4.1 vs. Model 4.3	χ^2^_diff_(1) = 18.72, *p* < .001						
	Model 4.2 vs. Model 4.4	χ^2^_diff_(1) = 16.50, *p* < .001						
	Model 4.3 vs. Model 4.4	χ^2^_diff_(1) = 6.78, *p* = .009						
	Model 4.4 with fixed vs. free coupling parameters	χ^2^_diff_(1) = 1.57, *p* = .210						

*Note*. Models 3 and 4 for the total sample included cognitive functioning and dummy coded intervention group as auxiliary variables for handling missing data.

*BIC* Bayesian Information Criteria, *CFI* Comparative Fit Index, *FH* functional health, *MHP* mental health problems, *SRH* self-rated health, *RMSEA* Root Mean Square Error of Approximation, *SRMR* Standardized Root Mean Square Residual

^*^ Finally selected model

**Fig. 1:**
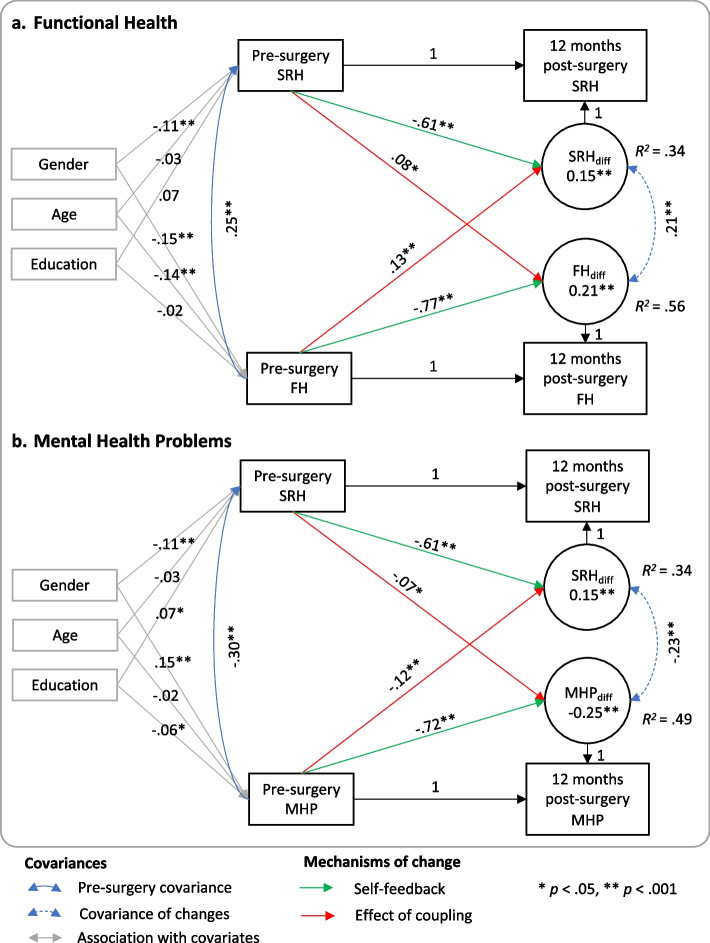
Bivariate latent change score models based on complete cases *Note*. Bivariate latent change score models for complete cases from pre-surgery assessment to 12 months post-surgery with bidirectional coupling effects and standardized coefficients. Results based on the total sample are presented as Supplementary Material, with showing the same pattern of results. FH = functional health; MHP = mental health problems; SRH = self-rated health. Note that higher scores of mental health problems indicate worse mental health, that is, a decrease in the score on mental health problems reflects an overall improvement of mental health, while an increase reflects a deterioration of mental health

In this model with bidirectional coupling effects (see Fig. 1 a.), we found a significant improvement of functional health, *FH*_*diff*_ = 0.21, 95% CI [0.16, 0.27], *p* < 0.001, and SRH, *SRH*_*diff*_ = 0.15, 95% CI [0.09, 0.20], *p* < 0.001, from pre-surgery to 12-months post-surgery. In line with hypothesis 1, pre-surgery functional health, *β* = -0.77,^1^ 95% CI [-0.86, -0.68], *p* < 0.001, and pre-surgery SRH, *β* = 0.08,^2^ 95% CI [0.03, 0.14], *p* = 0.001, significantly predicted changes in functional health. Worse pre-surgery functional health and—in accordance with hypothesis 6—better pre-surgery SRH were related to improvements of functional health and accounted together for 56% of the variance in pre-to-post surgery changes of functional health. Also, in line with hypothesis 2, pre-surgery SRH, *β* = -0.61, 95% CI [-0.66, -0.56], *p* < 0.001, and pre-surgery functional health, *β* = 0.13, 95% CI [0.07, 0.19], *p* < 0.001, predicted changes in SRH 12 months later, whereby worse pre-surgery SRH and better pre-surgery functional health were related to improvements of SRH and accounted for 34% of the variance in pre-to-post-surgery SRH changes. Speaking of unstandardized estimates, for respondents with average functional health and SRH, functional health increased by 2.53 (range: 0 – 100) along with an increase of 0.13 in SRH (range: 1 – 5), with both changes being positively associated (*r* = 0.21), that is, improvements of SRH were associated with improvements of functional health and vice versa. See Supplementary Material for illustrative calculations and estimates for the total sample.

Comparing a model constraining the coupling effects to be equal with a model allowing them to vary freely, revealed a significant difference in model fit, χ^2^_diff_(1) = 8.27, *p* = 0.004, indicating that pre-surgery functional health had a stronger influence on changes in SRH than vice versa.

### Model 2: Self-rated health and mental health problems

When comparing a unidirectional coupling model allowing for a path from pre-surgery SRH to changes in mental health problems with a model without coupling, the former was associated with a significantly improved model fit, χ^2^_diff_(1) = 8.97, *p* = 0.003 (see Table 3). The same applied to the unidirectional model allowing for a path from pre-surgery mental health problems to changes in SRH, χ^2^_diff_ (1) = 17.99, *p* < 0.001. Comparing these unidirectional models to the bidirectional model, the latter showed a better fit compared to both models, χ^2^_diff_(1) ≥ 7.24, *p*’s < 0.001 (see Table 3), and overall good fit as indicated by SRMR, CFI and RMSEA. Model comparisons based on the total sample showed the same pattern of results favoring a model with bidirectional coupling effects (see Supplementary Material).

In the model allowing for bidirectional coupling (see Fig. 1 b.), there were significant improvements in mental health, *MHP*_*diff*_ = -0.25, 95% CI [-0.30, -0.19], *p* < 0.001, and SRH, *SRH*_*diff*_ = 0.15, 95% CI [0.09, 0.20], *p* < 0.001. In line with hypothesis 3, pre-surgery mental health problems, *β* = -0.72, 95% CI [-0.77, -0.67], *p* < 0.001, and pre-surgery SRH, *β* = -0.07, 95% CI [-0.13, -0.02], *p* = 0.008, significantly predicted changes in mental health problems 12 months later. In accordance with hypothesis 6, better pre-surgery SRH and more severe pre-surgery mental health problems were related to improvements of mental health (i.e., decreases of mental health problems) and accounted together for 49% of the variance. Moreover, in accordance with hypothesis 4, pre-surgery SRH, *β* = -0.61, 95% CI [-0.66, -0.56], *p* < 0.001, and pre-surgery mental health problems, *β* = -0.12, 95% CI [-0.17, -0.06], *p* < 0.001, predicted changes in SRH at 12-months follow-up. Better pre-surgery mental health and worse SRH were associated with larger improvements and explained 34% of the variance in changes of SRH. With respect to unstandardized estimates, for respondents with average SRH and mental health problems, one would expect an increase of SRH by 0.13 (range: 1 – 5) and a decrease of mental health problems by -0.18 (range 0 – 3). Changes in SRH and mental health problems showed a significant negative association (*r* = -0.23), that is, improvements of SRH were associated with improvements of mental health and vice versa. Illustrative calculations for specific SRH and mental health constellations can be found in the Supplementary Material along with estimates for the total sample, which were highly similar. Comparing both coupling effects in size, these were not significantly different, χ^2^_diff_(1) = 2.25, *p* = 0.134. Thus, the association of pre-surgery SRH with changes in mental health problems was comparable to the association of pre-surgery mental health problems with changes in SRH.

## Discussion

The present study examined the interplay of self-rated health (SRH), functional health and self-reported mental health and their relative importance for predicting health changes after elective surgery, that is, a common health stressor in old age. Using prospective data from the PAWEL study, we found significant changes from pre-surgery to 12-months follow-up assessments for all health indicators. Our analyses provided evidence for an improvement of SRH, functional health and a reduction of mental health problems. Based on these findings, we employed BLCS models to further explore individual-level changes and their predictors. In line with our hypotheses, bidirectional models had superior fit compared to no coupling and unidirectional models. This finding emphasized the expectation that there is a substantial dynamic between SRH, functional and mental health.

In line with our hypotheses, changes of functional health from pre-surgery to 12-months follow-up were predicted by pre-surgery functional health but also by pre-surgery SRH, with worse pre-surgery functional health and better pre-surgery SRH being associated with larger improvements of functional health. Positive changes in SRH were associated with better pre-surgery functional health and worse pre-surgery SRH. However, the effect of pre-surgery SRH on changes in functional health was smaller than the inverse association of pre-surgery functional health with changes in SRH. Changes of SRH and functional health were positively correlated, that is, improvements of SRH were associated with improvements of functional health.

Findings for mental health were similar: Changes in mental health problems were associated with pre-surgery mental health problems and pre-surgery SRH, with worse pre-surgery mental health and better pre-surgery SRH being associated with larger decreases of mental health problems. Improvements of SRH were predicted by worse pre-surgery SRH and less severe pre-surgery mental health problems. In this model, the impact of pre-surgery SRH on changes in mental health problems and the inverse effect of pre-surgery mental health problems on changes in SRH were comparable in size. Changes of SRH were negatively correlated with changes in mental health problems, that is, an improvement of SRH was associated with a reduction of mental health problems and vice versa. Taken together, in line with our hypothesis, better pre-surgery SRH was associated with more favorable changes of other health indicators, that is, improvements of functional health and reduced mental health problems within the first year after elective surgery. These findings provide support for the potential role of SRH as physical resilience factor.

### Changes in self-rated health and functional health

We found SRH to increase from pre-to-post surgery. This may reflect successful recovery after surgery and may also point to the fact that some of the participants may have been at their worst health before undergoing surgery. At the same time, against the background of previous clinical studies that examined how SRH changed after significant health stressors, the overall significant increase of SRH we found is somewhat surprising. For example, SRH remained stable after total joint replacement [70] or even worsened after myocardial infarction [71]. Epidemiological longitudinal studies also showed that health stressors predicted decreases in SRH. However, especially in older participants, this decrease was not as strong as in younger individuals (e.g., [72]). Improvements of SRH had also been reported in older hospitalized samples (e.g., [31]). This can be explained by the fact that with increasing age, SRH and functional health indicators no longer correlate as strongly (e.g., [2, 73]). Even if their physical performance declines, older adults still rate their health as good [74].

In view of our study population with a mean age of 77.27 years, first, the significant increase in SRH could be founded in this age-related divergence. Second, in comparison to other studies like Perruccio et al. [70] and Benyamini et al. [71], we assessed SRH at a later follow-up timepoint (12 months vs. 3 and 6 months) which implies more time to recover from surgery. This time-related aspect could also have played a role in the evaluation of SRH. Third, our sample underwent planned surgical procedures. The intentional motivation to undergo a surgical procedure and recover from it is often driven by low pre-surgery health-related quality of life and the expectation to improve the current health status (e.g., [75]). Thus, the chance of improved post-surgery SRH is high if patients actually experience an improvement of their health status.

Findings from our BLCS models may help to shed light on the heterogeneous SRH changes and their predictors: Worse pre-surgery functional health and better pre-surgery SRH were associated with greater likelihood of negative SRH changes in the present study, while better pre-surgery functional health and worse pre-surgery SRH were associated with a higher likelihood of SRH improvements. The negative self-feedback effect, that is, better pre-surgery SRH is associated with more negative changes of SRH, may point to the fact that age-related health stressors like elective surgeries put a serious threat to general health especially for those with good pre-stressor SRH. At the same time, they may reflect that there is less room for potential SRH improvements in those with better pre-surgery SRH, making negative changes (e.g., from very good to good SRH) in those participants more likely.

Overall, we found a significant increase of functional health that could be considered as a positive consequence of the surgical intervention (e.g., better walking ability after orthopedic procedures) and represent the amelioration of a health issue. This may also have led to an improved perception of overall health compared to the situation before surgery and after recovery. As functional capabilities are key determinants (e.g., [76–78]) for the evaluation of SRH and an important predictor of SRH in older adults (e.g., [23]), an increase of SRH after surgery may be caused by a substantial improvement of functional health, which could be reflected in the positive association of changes in SRH and functional health in the BLCS model. Moreover, we found that pre-surgery functional health had a stronger influence on changes in SRH than vice versa. At the same time, our analyses yielded another negative self-feedback effect with lower pre-surgery functional health predicting improvements of functional health. That may imply that patients who have worse pre-surgery functional capabilities seem to benefit more from the surgical intervention, which may again point to a larger room for potential improvements in this group. However, also better pre-surgery SRH was associated with larger improvements of functional health. This finding is in line with results of epidemiological (e.g., [18]) and clinical studies (e.g., [31]) that identified fair or poor SRH as predictor of worse functional health in older adults. As we examined not only absolute outcome values as one of the first studies these findings suggest that worse SRH may not only represent a risk factor in older surgical patients for worse functional outcome per se, but also for potential negative *changes* in functional capabilities.

### Changes in self-rated health and mental health problems

Overall, we found a significant decrease of mental health problems in the current study. This contrasts with other studies regarding depression and anxiety after health stressors. For example, Murphy et al. [30] showed a substantial increase of depressive and anxiety symptoms after cardiac events. However, these differences may also point to the heterogenous quality of health stressors in older age, whereby cardiac events may constitute an acute grave event with long-lasting negative health consequences compared to an elective surgery that might result in a regain of health. In general, mental health problems may influence SRH in a negative way as epidemiological studies showed that depression and anxiety are strong predictors of poor SRH in old age (e.g., [79, 80]). Furthermore, mental well-being was the only health indicator to independently and consistently predict SRH after total joint replacement in a clinical study [14].

However, beyond previous studies focusing on unidirectional associations, our analyses provide insights on the interplay between changes in SRH and mental health. Worse pre-surgery SRH was associated with stronger increases in SRH (negative self-feedback) and more negative changes in mental health, while more severe mental health problems and better pre-surgery SRH were associated with a greater likelihood of negative SRH changes. Due to the overall increase in SRH, this could also imply that good pre-surgery SRH had a larger likelihood of remaining good whereas poor pre-surgery SRH is more likely to change over time. This is consistent with epidemiological studies that examined trajectories or response-shift in SRH in older adults in relation to their objective health status (e.g., [81, 82]).

In line with hypothesis 6, we found that better pre-surgery SRH was related to larger decreases of mental health problems. As we examined changes in mental health and not only absolute post-surgery values, we identified SRH not only as a predictor of mental health like other studies (e.g., [21, 29]), but also as a potential protecting factor against decreasing mental health after surgical interventions in older adults. These results are consistent with our expectation based on previous epidemiological and clinical studies (e.g., [21, 30]) and supports the notion that worse pre-surgery SRH is associated with less favorable changes of post-surgery mental health problems. Again, we found a negative self-feedback effect of mental health problems, that is, more severe pre-surgery mental health problems were associated with more positive pre-to-post surgery changes. This is line with findings on mental health changes in the general population during the COVID-19 pandemic [9, 83], a major societal-level health stressor, and might be again associated with greater room for improvements in those with severe pre-stressor mental health problems. On the other hand, stressor exposure may also result in short-term increases of mental health problems in those with good pre-stressor mental health, potentially reflecting mental reactivity to stressor exposure. In contrast to functional health, we found no difference in coupling effects between SRH and mental health problems, that is, SRH and mental health problems were equally important for their respective changes.

### Self-rated health as physical resilience factor?

Interpreting our findings in light of the discussion on physical resilience, our results provide preliminary support for the potential value of pre-health stressor SRH as patient-centered predictor of resilient responses in terms of more favorable adaptation processes in other health outcomes. Thus, assessing SRH in advance of surgical interventions to identify patients at risk for unfavorable outcomes, that is, functional decline and increase in mental health problems, may substantially improve surgical outcomes. In older surgical patients, functional decline often occurs after different types of surgery [84, 85], especially in nursing home residents [86, 87], and is associated with a higher risk of mortality [88, 89]. In addition, mental health problems like depression and anxiety are known to be associated with poorer outcomes after surgery like more severe pain and lower knee function [90, 91] or more readmissions, lower quality of life and higher mortality [92, 93]. To reduce such serious surgery-related consequences in older patients, individual pre-operative assessment has long been recommended [35, 94]. As time is limited in clinical routine, an efficient pre-surgery assessment is desirable. SRH may represent a composite indicator combining a number of functional and mental health characteristics that would otherwise have to be identified and evaluated by a more complex and time-consuming anamnesis (e.g., [95, 96]). Based on the results of our study and in line with suggestions of other studies (e.g., [97, 98]), we recommend including SRH as standard part of (pre-)admission assessment in older patients to identify patients at risk for unfavorable functional and mental health changes after elective surgery.

Future studies will have to examine the predictive value of SRH for more diverse age-related health stressors over longer periods and contrast its predictive power with other variables known to be associated with more favorable recovery processes and better health in older age (e.g., positive views on aging; [99, 100]). Moreover, future studies need to examine the nature of negative self-feedback effects found in this study and should examine whether these mainly mirror scale-related aspects (i.e., less room for positive changes) or a heightened risk of negative overall health changes in those with better pre-stressor health. From a resilience point of view, future research may examine the ability of SRH to predict not only decreases and increases of functional and mental health but also differential patterns of response. This may, on the long run, also allow for tailored timing of indicated health-promoting interventions in the context of age-related health stressors.

### Strengths and limitations

A main strength of our study is that we predicted changes—not only absolute values—in a longitudinal dataset with a large sample size that represents a broad range of surgical interventions, a very common health stressor, in old age. Assessing indicators at both time points, pre- and post-surgery, allowed us to make statements about individual-level deteriorations and improvements of SRH, functional and mental health as general health indicators. From a (physical) resilience point of view, the major strength of our study is the pre-stressor assessment allowing to examine the maintenance or regain of health from pre-to-post stressor [10, 13]. This kind of predictive value is of key importance from a research point of view, but also for clinicians as medical treatments like surgical interventions aim to improve and therefore change the state of health positively. Moreover, unlike other studies, we investigated bidirectional coupling effects which allowed us to examine the complex interplay of different health indicators and their relative importance for predicting changes in other health indicators. Thereby, our study may form a base for future studies investigating these relationships of longer periods with high-frequency assessments that will allow for disentangling constant and dynamic change process [101].

Besides these strengths, the current study also has some limitations that need to be considered when interpreting our results. First, we have a large amount of missing data. We addressed this issue by presenting results based on completers and the total sample separately, which lead to identical conclusions. However, we cannot exclude that the large number of missing data biased our results. A positive survival bias (i.e., inclusion of healthier participants) might have also resulted from our decision to exclude deceased individuals from our analytical sample. Second, our analyses do not allow for causal conclusions as they are based on an observational study and regression-based analyses, which are not suitable for individual-level prediction. Moreover, we cannot exclude that our findings may also be biased by unobserved confounding variables that impact on different health indicators. Third, data were collected for another purpose than our research interest, so the measurement timing and use of questionnaires is not ideal. For example, we lacked information about SRH at day of discharge. Such information would be useful for modeling the dynamics of recovery processes at higher frequency and to identify different patterns of recovery along with their predictors. Psychological resilience research may provide an inspiration for such study designs (e.g., [8, 9]). Fourth, respondents included in our study were relatively healthy in terms of high functional health and low mental health problems (but showed medium SRH levels). This may point to positive selection effects during sample recruitment, that is, a larger chance of healthier patients to participate in the study. Our model findings might have been impacted by this positive selection bias (e.g., strong negative self-feedback may mirror a smaller number of patients having much room left for positive changes). The reliance on self-report data for our study is both a limitation and a strength. On the one hand, findings for clinician-made diagnoses might have yielded differential results [102]; on the other hand, there is an increasing interest in patient-centered medicine and outcome assessment [103] for which self-reports as obtained in our study are essential. Thus, the findings of the current study improve our knowledge about pre-surgery SRH as a predictor of changes in functional and mental health after elective surgery in old age and their bidirectional relationships.

## Conclusions

Taken together, we demonstrated that pre-surgery SRH might have the potential to be used as predictor of adaptation processes after common age-related health stressors like elective surgeries. While good SRH might predict resilient responses, that is, the maintenance or fast regain of functional and mental health after exposure to health stressors, poor SRH may point to a heightened risk for less favorable responses, that is, less positive changes in functional and mental health. Vice versa, worse pre-surgery functional and mental health might be viewed as risk factors for unfavorable post-surgery changes of SRH and therefore reduced health-related quality of life. Due to the high importance of functional and mental health outcomes for older patients after surgery (e.g., [34, 35]), our findings contribute to a deeper understanding of changes in central health indicators in a clinical context. Nevertheless, more longitudinal clinical studies with a larger number of assessments are needed in the future to investigate the predictive value of SRH for multidimensional health trajectories.

## Supplementary Information

**Additional file 1:**

## Data Availability

The datasets used and/or analyzed during the current study are available from the corresponding author on reasonable request.

## Abbreviations

BI: Barthel Index
BLCS: Bivariate latent change score (models)
FH: Functional health
MHP: Mental health problems
PHQ-4: 4-Item Patient Health Questionnaire
SD: Standard deviation
SRH: Self-rated health

## Footnotes

1 That is, lower levels (i.e., worse) of pre-surgery functional health are associated with larger increases (i.e., improvements) in functional health.

2 That is, higher levels (i.e., better) of pre-surgery SRH are associated with larger increases (i.e., improvements) in functional health.
