# Supplementary material for: The relationship between pre-surgery self-rated health and changes in functional and mental health in older adults: insights from a prospective observational study
Source: BMC Geriatr. 2023 Mar 31;23:203. doi: 10.1186/s12877-023-03861-x (PMC10064967; doi:10.1186/s12877-023-03861-x)
Supplement: Supplementary file 1 — Additional file 1: [file 12877_2023_3861_MOESM1_ESM.pdf]

Online Supplementary Material

Title: The relationship between pre-surgery self-rated health and long-term changes in functional and mental health problems in older adults: insights from a prospective observational study

Authors: Mennig, E. F., Schäfer, S. K., Eschweiler, G. W., Rapp, M. A., Thomas, C., Wurm, S.

Details on modeling choice

We favored BLSC models above other models available to examine longitudinal changes [e.g., latent growth curve models, (random intercept) cross-lagged panel models] based on theoretical considerations. First, we were specifically interested in the prediction of changes and bidirectional coupling effects on changes rather than in the prediction of observed or residualized scores (1). Second, in line with the concept of BLCS models, we assume changes in SRH and health indicators to constitute a dynamic process, with scores at a given time being conceptualized as a function of previous scores (2). As we examine health indicators in the context of a health stressor, we assume that there is a constant process of recovery (i.e., constant change), however, with specific changes depending on previous health status (i.e., proportional change). From a resilience point of view on recovery this is plausible (3) as individual changes may depend on previous health status and are likely to vary between an initial phase of adaptation and later phases of recovery. Although, we cannot disentangle constant and proportional change within the current study, we believe that the study of these change components will provide further insights into the complex interplay between SRH, functional and mental health following health events (4). We acknowledge that there are latent growth curve model equivalents for BLSC. However, we believe that future research will benefit from modeling dual change of SRH, functional and mental health. Such models were found to be harder to fit using a latent growth curve approach (2).

Figure S1. Schematic illustration of the model

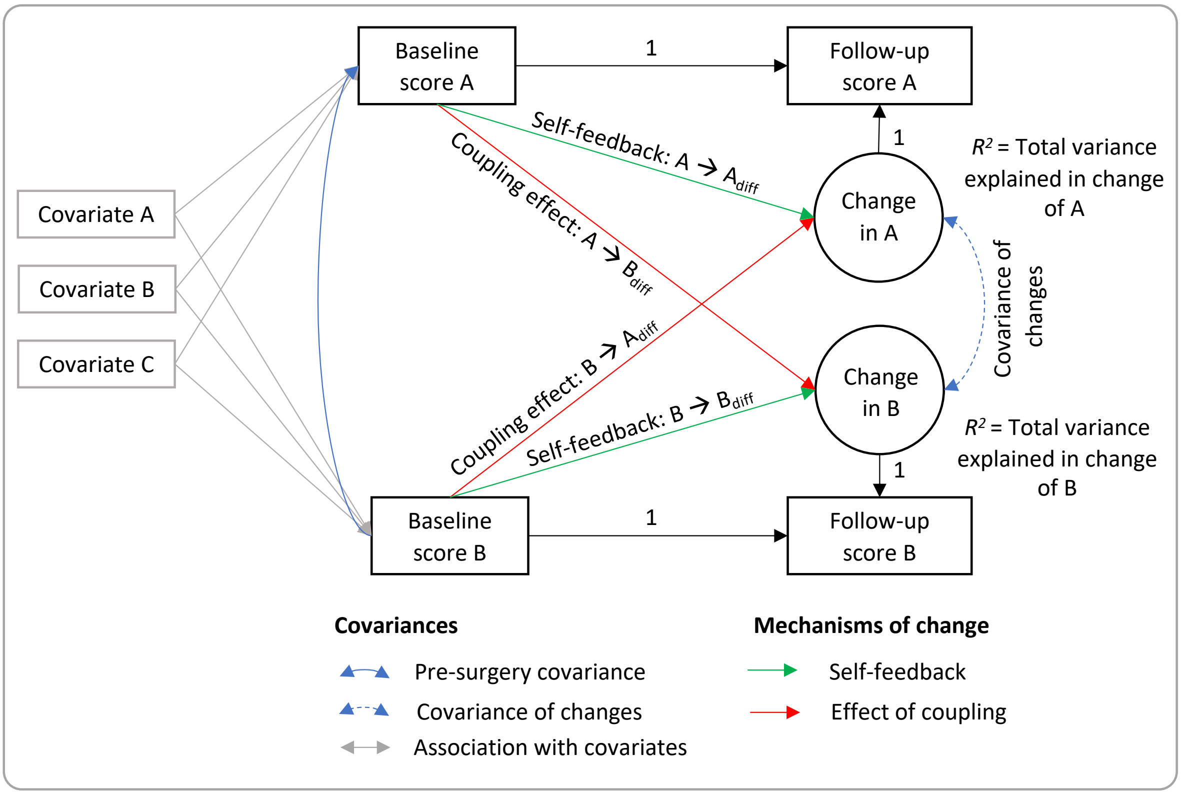

Note. Schematic illustration of the bivariate latent change score models used in this study. The illustration was adapted and amended from the tutorial paper of Kievit et al. (5). For our models, raw data has been centered relative to baseline means of the respective outcome, thus, positive changes (A_{diff} or $B_{diff} > 0$) in the respective construct indicate that individuals with average baseline levels in construct A and B have a greater likelihood of experiencing increases of the respective construct, while negative changes (A_{diff} or $B_{diff} < 0$) indicate that people with average levels have a greater likelihood to experience decreases. Thus, the significance test of the intercept of the respective change scores indicates whether there is a significant change (i.e., increase or decrease) in the respective outcome at average baseline levels.

Model comparisons: No coupling model: Both red paths fixed to zero. Unidirectional coupling models: Only one red path is estimated freely, the other is fixed to zero. Bidirectional coupling model: Both red paths are estimated freely.

Model selection based on the total sample

1. Functional status

No coupling model vs. unidirectional coupling from pre-surgery SRH to changes in functional status: $\chi^2_{diff}(1) = 9.54$, $p = .002$

No coupling model vs. unidirectional coupling from pre-surgery functional status to changes in SRH: $\chi^2_{diff}(1) = 14.43$, $p < .001$

Unidirectional coupling from pre-surgery SRH to changes in functional status vs. bidirectional coupling effects: $\chi^2_{diff}(1) = 13.13$, $p < .001$

Unidirectional coupling from pre-surgery functional status to changes in SRH vs. bidirectional coupling effects: $\chi^2_{diff}(1) = 8.17$, $p = .004$

2. Mental health problems

No coupling model vs. unidirectional coupling from pre-surgery SRH to changes in mental health problems: $\chi^2_{diff}(1) = 8.49$, $p = .004$

No coupling model vs. unidirectional coupling from pre-surgery mental health problems to changes in SRH: $\chi^2_{diff}(1) = 18.72$, $p < .001$

Unidirectional coupling from pre-surgery SRH to changes in mental health problems vs. bidirectional coupling effects: $\chi^2_{diff}(1) = 16.50$, $p < .001$

Unidirectional coupling from pre-surgery mental health problems to changes in SRH vs. bidirectional coupling effects: $\chi^2_{diff}(1) = 6.78$, $p = .009$

Figure S2. Bivariate latent change score models based on the total sample

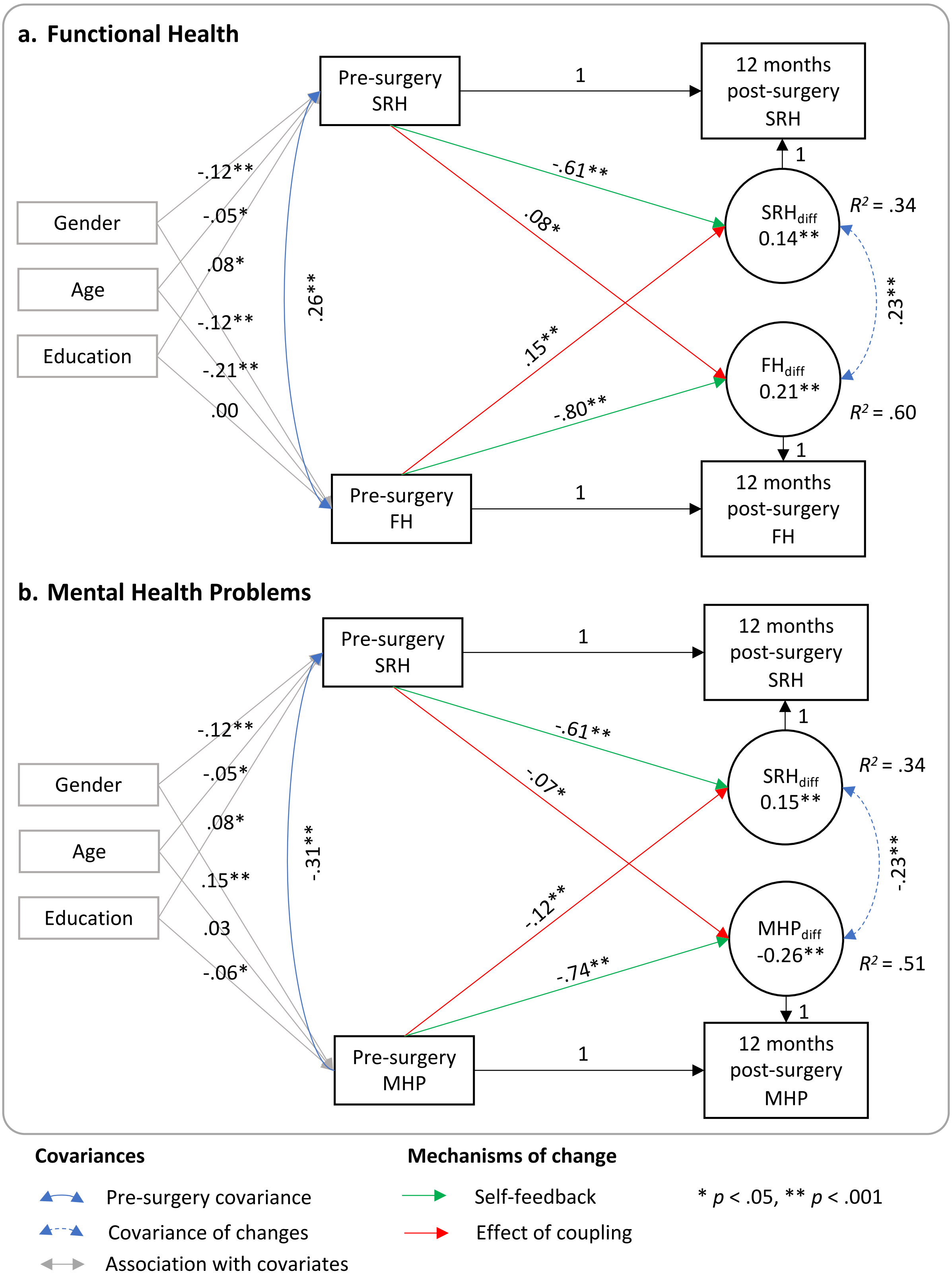

Note. Bivariate change score models for the total sample from pre-surgery assessment to 12 months post-surgery with bidirectional coupling effects and standardized coefficients. Results largely resample those based on complete cases. FH = functional health; MHP = mental health problems; SRH = self-rated health.

Illustrative examples of changes in health indicators

Table S1. Summary of predicted changes based on bivariate change models

		Self-rated health			Self-rated health
Functional health	low	+ 0.06 ↑	Mental health problems	low	+ 0.20 ↑↑
	high	+ 0.23 ↑↑		high	+ 0.03 ↑
Self-rated health	low	+ 0.72 ↑	Self-rated health	low	+ 0.68 ↑
	high	- 0.37 ↓		high	- 0.41 ↓
		Functional status			Mental health problems
Functional health	low	+ 11.74 ↑	Mental health problems	low	+ 0.14 ↑
	high	-1.45 ↓		high	- 0.72 ↓
Self-rated health	low	+ 1.52 ↑	Self-rated health	low	- 0.13 ↓
	high	+ 3.50 ↑↑		high	- 0.24 ↓↓

Note. Changes were predicted based on the equations provided by Jajodia (6). ↓= decrease (i.e., a deterioration of functional health and self-rated health and an improvement of mental health by reduced mental health problems); ↑= increase (i.e., an improvement of functional health and self-rated health and a deterioration of mental health by an increase of mental health problems); ↓↓= stronger decrease (i.e., a stronger deterioration of functional health and self-rated health and a stronger improvement of mental health by reduced mental health problems); ↑↑= stronger increase (i.e., a stronger improvement of functional health and self-rated health and a stronger deterioration of mental health by an increase of mental health problems). Differences may arise from anticipated decreases vs. increases or from decreases/increases of different strength.

Changes refer to calculations varying the respective predictor when the other predictor was set to average. For example, when pre-surgery functional health was low (i.e., 1 *SD* below mean) and pre-surgery self-rated health was average, one would expect an increase of +0.06 in self-rated health, which is numerically smaller than the expected decrease for high pre-surgery functional health (+0.23). Thus, in our bivariate change model better pre-surgery functional health was associated with larger improvements in self-rated health. Low or high levels of each variable present minimal or maximal values for the respective variable or values at mean levels ± 1 *SD*.

Details on the PAWEL study

The acronym PAWEL stands for "Patient safety, cost-effectiveness, and quality of life: reduction of delirium risk and postoperative cognitive dysfunction after elective procedures in older adults" (7). The study was conducted in five medical centers in the southwest of Germany from 2017 to 2021. The project investigated the extent to which a cross-sectoral multimodal delirium prevention intervention reduces delirium prevalence and cognitive deficits after elective surgery in patients over 70 years of age (8) and whether it is cost-effective. Further aims were to develop a valid screening and testing instrument for delirium and postoperative cognitive deficits (POCD), to implement a delirium risk score (9), to outline a training concept for the intervention team, and to describe the cross-sectoral and interprofessional pathways of patients.

References

1. Orth U, Clark DA, Donnellan MB, Robins RW. Testing prospective effects in longitudinal research: Comparing seven competing cross-lagged models. *J Pers Soc Psychol*. 2021;120(4):1013-34.
2. Serang S, Grimm KJ, Zhang Z. On the correspondence between the latent growth curve and latent change score models. *Struct Equ Modeling*. 2019;26(4):623-35.
3. Gijzel SMW, Whitson HE, van de Leemput IA, Scheffer M, van Asselt D, Rector JL, et al. Resilience in Clinical Care: Getting a Grip on the Recovery Potential of Older Adults. *J Am Geriatr Soc*. 2019;67(12):2650-7.
4. Schäfer SK, Fleischmann R, von Sarnowski B, Bläsing D, Flöel A, Wurm S. Relationship between trajectories of post-stroke disability and self-rated health (NeuroAdapt): protocol for a prospective observational study. *BMJ Open*. 2021;11(6):e049944.
5. Kievit RA, Brandmaier AM, Ziegler G, van Harmelen AL, de Mooij SMM, Moutoussis M, et al. Developmental cognitive neuroscience using latent change score models: A tutorial and applications. *Dev Cogn Neurosci*. 2018;33:99-117.
6. Jajodia A. Dynamic structural equation models of change. Longitudinal data analysis: A practical guide for researchers in aging, health, and social sciences. Multivariate application series. New York, NY, US: Routledge/Taylor & Francis Group; 2012. p. 291-328.
7. Sánchez A, Thomas C, Deeken F, Wagner S, Klöppel S, Kentischer F, et al. Patient safety, cost-effectiveness, and quality of life: reduction of delirium risk and postoperative cognitive dysfunction after elective procedures in older adults-study protocol for a stepped-wedge cluster randomized trial (PAWEL Study). *Trials*. 2019;20(1):71.
8. Deeken F, Sánchez A, Rapp MA, Denkinger M, Brefka S, Spank J, et al. Outcomes of a Delirium Prevention Program in Older Persons After Elective Surgery: A Stepped-Wedge Cluster Randomized Clinical Trial. *JAMA Surgery*. 2021:e216370-e.

9. Eschweiler GW, Czornik M, Herrmann ML, Knauer YP, Forkavets O, von Arnim CAF, et al. Presurgical Screening Improves Risk Prediction for Delirium in Elective Surgery of Older Patients: The PAWEL RISK Study. Front Aging Neurosci. 2021;13:679933.
